# KLVFF Conjugated Curcumin Microemulsion-Based Hydrogel for Transnasal Route: Formulation Development, Optimization, Physicochemical Characterization, and Ex Vivo Evaluation

**DOI:** 10.3390/gels9080610

**Published:** 2023-07-28

**Authors:** Rungsinee Phongpradist, Jutamas Jiaranaikulwanitch, Kriangkrai Thongkorn, Suree Lekawanvijit, Sasithorn Sirilun, Chuda Chittasupho, Worrapan Poomanee

**Affiliations:** 1 Department of Pharmaceutical Sciences, Faculty of Pharmacy, Chiang Mai University, Chiang Mai 50200, Thailand; rungsinee.p@cmu.ac.th (R.P.); jutamas.jia@cmu.ac.th (J.J.); sasithorn.s@cmu.ac.th (S.S.); chuda.c@cmu.ac.th (C.C.); 2 Department of Companion Animals and Wildlife Clinic, Faculty of Veterinary Medicine, Chiang Mai University, Chiang Mai 50100, Thailand; kriangkrai.th@cmu.ac.th; 3 Department of Pathology, Faculty of Medicine, Chiang Mai University, Chiang Mai 50200, Thailand; suree.lek@cmu.ac.th

**Keywords:** microemulsion-based hydrogel, curcumin, KLVFF, central composite design, microemulsion, transnasal, Alzheimer

## Abstract

Curcumin is a potent natural compound used to treat Alzheimer’s disease (AD). However, the clinical usefulness of curcumin to treat AD is restricted by its low oral bioavailability and difficulty permeating the blood-brain barrier. To overcome such drawbacks, various alternative strategies have been explored, including the transnasal route. However, rapid mucociliary clearance in the nasal cavity is a major hindrance to drug delivery. Thus, designing a delivery system for curcumin to lengthen the contact period between the drug and nasal mucosa must be employed. This study describes the optimization of KLVFF conjugated curcumin microemulsion-base hydrogel (KCMEG) to formulate a prototype transnasal preparation using the response surface method to improve a mucoadhesive property. A central composite design was employed to optimize and evaluate two influencing factors: the concentration of carbopol 940 and the percentage of KLVFF conjugated curcumin microemulsion (KCME). The physicochemical properties, anti-cholinesterase activity, and anti-aggregation activities of KCME were investigated in this study. The studied factors, in terms of main and interaction effects, significantly (*p* < 0.05) influenced hardness and adhesiveness. The optimized KCMEG was evaluated for pH, spreadability, and mucoadhesive properties. Ex vivo nasal ciliotoxicity to optimize KCMEG was performed through the porcine nasal mucosa. KCME was transparent, with a mean globule size of 70.8 ± 3.4 nm and a pH of 5.80 ± 0.02. The optimized KCMEG containing 2% carbopol 940 showed higher in vitro mucoadhesive potential (9.67 ± 0.13 min) compared with microemulsion and was also found to be free from nasal ciliotoxicity during histopathologic evaluation of the porcine nasal mucosa. The result revealed that both the concentration of carbopol 940 and the percentage of KCME play a crucial role in mucoadhesive properties. In conclusion, incorporating a mucoadhesive agent in a microemulsion can increase the retention time of the formulation, leading to enhanced brain delivery of the drug. Findings from the investigation revealed that KCMEG has the potential to constitute a promising approach to treating AD via transnasal administration.

## 1. Introduction

Alzheimer’s disease (AD) is a disorder that causes degeneration of the cells in the brain, and it is the main cause of dementia marked by declines in cognitive abilities and behavioral alterations [1]. Swallowing impairments in Alzheimer’s patients are the leading cause of progressive reductions in solid and liquid drug or food intake [2]. Thus, solid dosage forms such as tablets or capsules used to treat AD can pose problematic issues for patients, particularly those with late-stage AD who may experience problems associated with swallowing [1]. Moreover, conventional oral administration presents relevant limitations such as bioavailability, rapid metabolism, limited brain exposure, and even some undesirable side effects [3]. These factors led to the development of drug formulations that are not taken orally. The parenteral route also limits the accessibility of drugs during blood circulation to the brain due to blood-brain barriers (BBB) [4]. Moreover, systemic clearance also affects the bioavailability of drugs for parenteral administration. However, drugs can be directly delivered to the brain via intrathecal drug administration but may result in operative and postoperative complications such as hemorrhage or catheter-related infection [5]. Hence, an alternative route of administration should be preferable. Transnasal dosage form has been strongly demonstrated as a potential carrier for drugs to directly transport in the central nervous system (CNS) via the olfactory and trigeminal pathways [6,7]. Administration through the nasal mucosa promotes faster and higher levels of drug absorption. This is primarily attributed to the nasal cavity’s high permeability, low enzymatic environment, and rich vasculature [8,9,10]. Transnasal administration has recently received attention due to several advantages, including direct access to medications from the nasal cavity to the central nervous system, noninvasive administration, simplicity of use for self-medication, and improved patient compliance [11,12]. Additionally, the blood-brain barrier and hepatic first-pass metabolism, the principal barriers to oral medication administration, can be avoided with transnasal delivery to the brain [13,14]. This suggests that the transnasal administration route has the potential to maintain therapeutic drug concentrations in the brain while minimizing the required drug doses. Due to the higher permeability of the nasal mucosa compared to the blood-brain barrier (BBB), transnasal drug delivery is gaining interest as an effective method for delivering peptides and protein drugs. These types of drugs are particularly challenging to deliver to the brain through systemic circulation [15,16,17]. For the treatment of AD, several transnasal drug delivery systems have been investigated for their potential to transport drugs directly to the brain, e.g., microemulsion [18], noisome [19], and nanostructured lipid carriers [20]. However, transnasal delivery is constrained by poor drug diffusion through the nasal mucosa, resulting in insufficient concentrations of drug delivery to the desired target region and the inability to accomplish the therapeutic effect [12]. In addition, nasal mucociliary clearance is a natural defense mechanism that limits nasal administration [11]. Hence, improving drug absorption and avoiding fast nasal drainage are key strategies for developing formulations; therefore, formulations considered for transnasal administration should lengthen the contact period between the drug and nasal mucosa [11,21]. Transnasal drug delivery comes with several other disadvantages. These include the possibility of active drug degradation by the enzymes found in the nasal mucosa [22], reduced drug absorption for drugs with higher molecular weight [23], the potential for drug-induced irritation of the nasal mucosa, the risk of mucosal damage from frequent use, and significant changes in drug deposition caused by local nasal infections like the common cold [6,17].

Curcumin, the major polyphenol found in the rhizome of *Curcuma longa,* is widely recognized for its potent therapeutic properties in the treatment of Alzheimer’s disease (AD) [24]. However, the clinical usefulness of curcumin to treat AD is restricted by its low water solubility (11 ng/mL); thus, it is rapidly eliminated from the body and has low oral bioavailability [25]. Moreover, the use of curcumin faces obstacles due to its difficulty in permeating the blood-brain barrier. In order to enhance the biological and pharmacological activity of curcumin, a number of drug delivery systems offered by the transnasal route are utilized to deal with these limitations, such as nanocrystals [26] and nanoemulsions [27].

Over the decades, microemulsions (ME) have attained special attention as a colloidal drug delivery system for transnasal administration owing to their effectiveness in permeating across the nasal mucosa, bypassing the blood-brain barrier, and delivering the drugs at adequate concentrations to the brain [28]. Moreover, ME have many characteristics that make them attractive for transnasal drug delivery, including their ease of preparation due to spontaneous formation, thermodynamic stability, transparent and elegant appearance, increased drug loading and enhanced penetration through the biological membranes, increased bioavailability, and less inter- and intra-individual variability in drug pharmacokinetics [21,29]. Although the transnasal route presents numerous advantages, an inherent disadvantage is mucociliary clearance, which reduces the time allowed for drug absorption due to the fast clearance of the administered formulation from the nasal cavity [30]. Thus, to deliver the drug using the transnasal route, the formulation should be rapidly transported across the nasal mucosa and retained on the mucosa for a prolonged time before being cleared by nasal mucociliary clearance [30]. Various formulation factors, such as formulation viscosity, have been found to influence drug absorption during transnasal delivery [6]. A study conducted by Jansson et al. revealed that increasing the viscosity of the formulation prolongs the duration of contact between the drug and nasal mucosa, leading to enhanced drug absorption [31]. Consequently, a higher amount of the drug is absorbed into the system. Related literature has reported that improved drug absorption through the nasal mucosa could be achieved using mucoadhesive agents, whereby their interaction with mucin takes place and results in prolonged contact time between the applied formulation and the nasal mucosa [29,32,33]. The results of Barakat et al. revealed that after transnasal administration, the mucoadhesive formulation demonstrated a higher concentration of carbamazepine in the brain than that in plasma, indicating that extending the residence duration on the mucosa enhanced drug absorption [21,34]. Mucoadhesive formulations can be produced by incorporating mucoadhesive agents, which are substances that aid in adhering to a mucosal membrane, leading to continuous drug delivery to the brain [35]. There are various mucoadhesive formulations that have been reported, such as gel, tablet, ointment, and film agents [36]. Extensive research has been carried out on the delivery of drugs via the intranasal route using mucoadhesive microemulsion for the treatment of AD [18,30,37,38]. One notable example is the study of Jogani et al., which demonstrated that intranasal administration of mucoadhesive microemulsion loaded with tacrine, an Alzheimer’s therapeutic drug, showed that the brain bioavailability was more than twice that of intranasal tacrine solution [39]. As mentioned above, mucoadhesive drug delivery systems prolong the retention time at the site of application and improve bioavailability; thus, concomitantly formulating ME would be beneficial using mucoadhesive agents as mucoadhesive ME for the transnasal route. 

In order to fabricate an optimized formulation presenting desirable mucoadhesive properties, many variables may influence the product’s performance. Design of Experiment (DOE) has been effectively employed to statistically evaluate the significance of multiple independent variables to response variables by a small number of experiments at once, together with the optimal levels of the response variables. Eventually, DOE can generate mathematical models of the optimized process used to predict the formulation’s performance under different conditions. Not only precise information but also reduced experimental time and cost are the advantages of DOE over traditional experimental methods [40]. Central composite design (CCD) is one of the powerful experimental designs of DOE used in response surface methodology (RSM), consisting of a full factorial design plus center and axial points, which allows fitting a second-order polynomial model and more accurate estimation of the response variables [41].

In our related study, we have formulated a curcumin-loaded microemulsion coupled with the KVLFF peptide (KCME) as dual targeting to treat Alzheimer’s disease using the transnasal route [32]. Although we successfully developed KCME, it was highlighted that KCME characteristics should be improved to facilitate the attachment of KCME in the nasal cavity [30]. Notably in the literature, muco-adhesive ME can extend their time in contact with the mucosa and increase the viscosity of their formulations [21,33]. Hence, the strategy of this current study was to synthesize KCME-based hydrogel (KCMEG) to enhance the formulation’s muco-adhesiveness. This study aimed to investigate the pharmacologic effects of KCME on inhibiting acetylcholinesterase and amyloid aggregation. KCMEG was optimized and evaluated regarding significant factors on muco-adhesive properties in terms of texture profiles of KCMEG using an applied aspect as central composite design (CCD). Optimized KCMEG was further evaluated for physicochemical properties and ex vivo nasal cytotoxicity to ensure the feasibility of KCMEG for transnasal administration.

## 2. Results and Discussion

### 2.1. Physicochemical Characterization of ME and Microemulsion-Based Hydrogel

The physicochemical properties of the formulation play an essential role in predicting the system’s behavior and stability. Small globule sizes are expected to present a high permeation rate for nasal administration [42]. The pH value should be close to nasal secretion to avoid nasal irritation for patients [42]. Thus, globule size, pH, polydisperse index (PDI), % transmittance, zeta potential, and spreadability of KCME are shown in Table 1. KCME exhibited a small globule size of 70.8 ± 3.4 nm, and the pH value of KCME was in the range of nasal secretion (4.5 to 6.5), indicating less chance for irritancy in the nasal mucosa [7,42]. The percentage of transmittance was greater than 97%, confirming the system’s transparency. Based on the results, it could be concluded that KCME was expected to have good physical stability and less irritancy for nasal administration. Thus, KCME is optimal for preparing microemulsion-based hydrogels.

**Figure 1:**
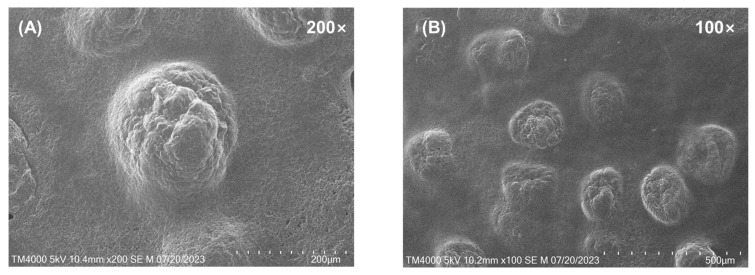
Scanning electron microscopy (SEM) images of KCMEG at 200× (**A**) and 100× (**B**).

The pH value of optimized KCMEG from CCD was 4.77 ± 0.23, which is in the range of the normal pH of nasal fluid, indicating less irritation for nasal use. Spreadability is a criterion for the topical dosage form to investigate the ease of application [43]. Interestingly, the diameter of the spread area of KCMEG was less than that of KCME (Table 2), which was probably due to the effect of their viscosity, which was reported as inversely proportional to spreadability [43]. To evaluate the morphology of optimized KCMEG, photomicrographs were taken at different magnifications (100× and 200×) using scanning electron microscopy (SEM), as shown in Figure 1. KCMEG was relatively uniform in size and spherical in shape.

### 2.2. Fourier Transform Infrared (FTIR) Spectroscopy

**Figure 2:**
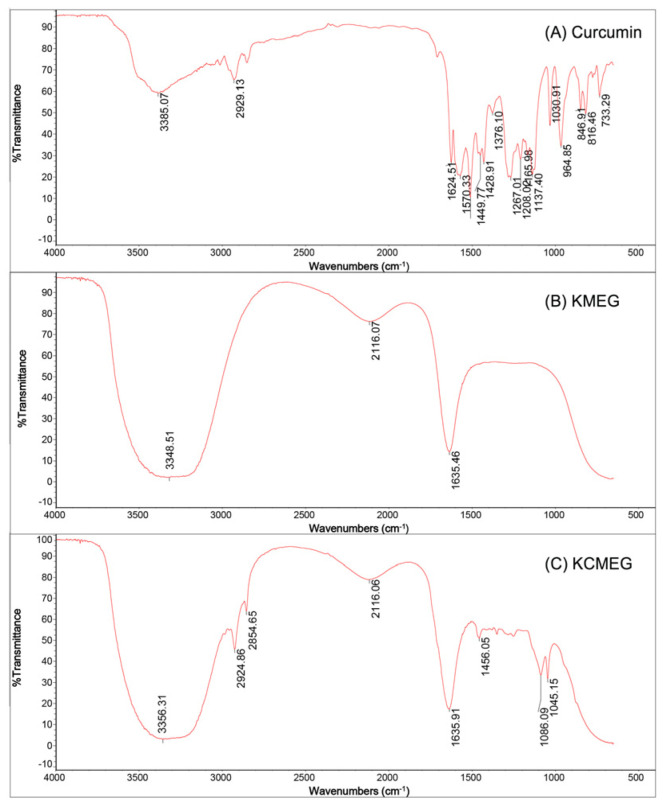
FTIR spectra of curcumin (**A**), KMEG (**B**) and KCMEG (**C**).

The IR spectra of curcumin, blank KLVFF-Pluronic F127 ME hydrogel (KMEG), and KCMEG in the frequency region from 400 to 4000 cm^−1^ are illustrated in Figure 2. The curcumin spectrum shows stretching vibrations of OH from the phenolic hydroxyl group at 3385.07 cm^−1^, at 2929.13 and 2854.65 cm^−1^ representing C-H stretching, and at 1624.51 cm^−1^ is the so-called C=C symmetric aromatic ring stretching. The FTIR spectrum of curcumin appeared similar to that reported in the literature [44]. The IR spectra of KCMEG differed significantly from those of curcumin but were very similar to those of KMEG. The FTIR spectra of carbopol in KMEG and KCMEG (Figure 2B,C) showed the carbonyl stretch band at 1635 cm^−1^ which was well in agreement with reported data [45,46]. However, KCMEG exhibited peaks at 2294 cm^−1^ and 2845.65 cm^−1^, which are the characteristic peaks of curcumin, indicating the existence of curcumin in KCMEG. In the case of curcumin in KCMEG, the characteristic bands of curcumin have either disappeared or had their intensity reduced due to restrictions within the formulation matrix. Based on the results, it was revealed that there were no interactions between curcumin and excipients. The reduced intensity and minor shifting of the characteristic peaks of curcumin might be attributed to some bonds, such as Van der Wall forces, hydrogen bonds, or dipole interactions, between curcumin and other excipients [47]. No additional peak was observed in the IR spectra of KCMEG, indicating the absence of any possible interaction between the drug and the formulation components used [48].

### 2.3. Anticholinesterase Activity

**Figure 3:**
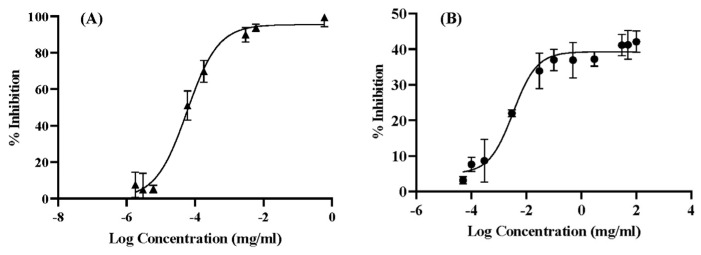
Dose-response curve of Galantamine HCl (**A**) and KCME (**B**) for anti-AChE.

Cholinergic neurotransmission plays a central role in impaired cognitive function in AD [49]. One of the most effective treatment strategies against AD is to enhance the acetylcholine level in the brain using acetylcholinesterase (AChE) inhibitors [50,51]. To evaluate the potential of KCME to treat AD, the inhibitory effect on AChE activities must be quantified. The dose-response curves for the anti-AChE action of KCME are depicted in Figure 3. The IC_50_ value of KCME was calculated to be 3.30 µg/mL, while that of galantamine hydrochloride was 0.065 µg/mL. Compared with a related study showing an IC_50_ value of 67.69 µM (24.68 mg/mL) for curcumin [52] and the reports of the improved solubility of curcumin by microemulsion [53,54], it would be reasonable to conclude that KCME has the potential to constitute an effective treatment strategy for AD treatment. Nonetheless, in vivo and clinical research needs to be undertaken to offer convincing evidence.

### 2.4. Anti-Aggregation of Beta-Amyloid

**Figure 4:**
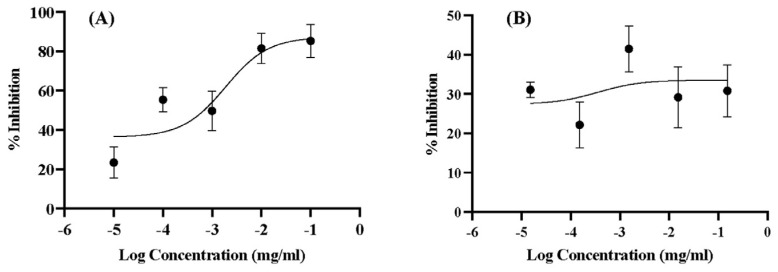
Dose-esponse curve of curcumin (**A**) and KCME (**B**) for anti-aggregation of amyloid beta.

The accumulation of aggregated Aβ with an extensive β-sheet structure forming senile plaques in the brain is one of the pathologic hallmarks of AD [55]. Therapeutic approaches targeting inhibiting Aβ aggregation are one promising treatment to overcome AD. Thus, the inhibitory effect on Aβ aggregation of KCME should be investigated. A thioflavin T assay revealed the inhibition of β-amyloid (Aβ) aggregation of KCME with a calculated IC_50_ value of 0.36 µg/mL, which was 5.18-fold stronger than that of the positive control, curcumin (1.87 µg/mL), as shown in Figure 4. This phenomenon might be explained by the KLVFF peptide and curcumin in KCME. The KLVFF peptide corresponds to the Aβ_16–20_ fragment, which plays a critical role in inhibiting Aβ aggregation by binding the confined region of the Aβ strand and interfering with the assembling and aggregation of Aβ from the attachment of neighboring Aβ by hydrogen bonding and hydrophobic interactions [55,56]. Additionally, curcumin has been demonstrated to inhibit aggregation and promote disaggregation of fibrillar Aβ in vivo and in vitro [57,58]. The mechanism of curcumin’s action on anti-Aβ aggregation may be due to the hydrophobicity of curcumin or the interactions between the keto or enol rings of curcumin and aromatic rings of Aβ dimers destabilized by the attractions requisite for forming beta-sheets in Aβ plaques [55]. In addition, hydroxyl groups of curcumin interact with polar pockets of the Aβ peptide, destabilizing beta-sheets [58]. The result demonstrates the potential of KCME to inhibit Aβ aggregation and indicates the potential of KCME to treat AD. However, the synergistic reaction between KLVFF and curcumin in KCME should be tentatively explained and requires more confirmation by mathematical modeling and experiments.

### 2.5. Formulation Optimization and Data Analysis

Because mucoadhesiveness of the formulation plays a crucial role in the therapeutic outcome of transnasal formulation, factors involving these characteristics in terms of hardness and adhesiveness as response variables were evaluated by CCD, implying statistical correlations between studied factors and responses using simultaneous determination of various factors at one time [59].

The experimental data of the two response variables, including hardness (Y_1_) and adhesiveness (Y_2_), as shown in Table 3, were analyzed to establish the best statistical models using Design-Expert^®^ Software (Version 10.0; Stat-Ease Inc.). The obtained models showed a high coefficient of determination (*R*^2^) in the range of 0.9010 to 0.9464, implying that more than 90% of the variations of the two responses could be explained using the models. The software statistically generates the *F*-value and *p*-value according to the calculations of the sum of squares and mean squares of the final model, including significant terms and residuals, which are non-significant terms excluded from the final model. In addition, as shown in Table 3, a high *F*-value and a small *p*-value for each term indicate a highly significant effect on the response variables. The regression coefficient of each term was positively correlated to its *F*-value. Independent variables were rationally included by considering a *p*-value less than 0.05 to generate the significant model (*p* < 0.05) for statistically describing the correlation between two studied factors and hardness (Y_1_) as follows:

Y_1_ = 7.06 + 0.129X_1_ − 11.82X_2_ − 6.26X_1_X_2_

**Figure 5:**
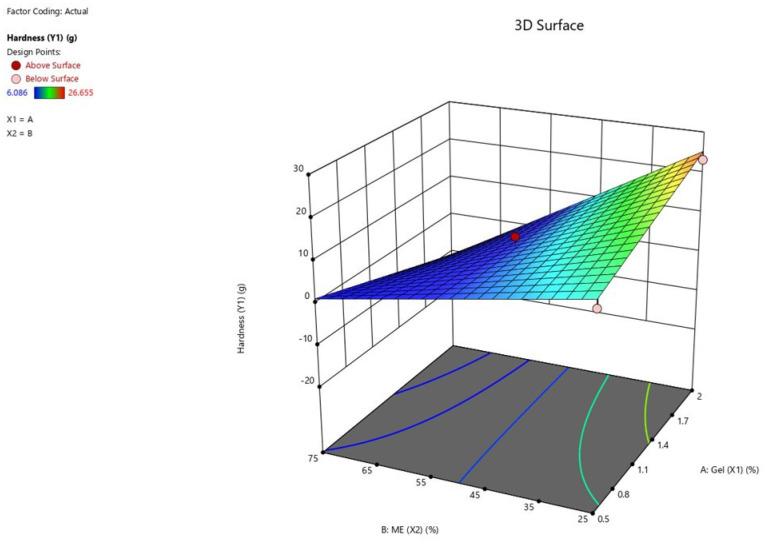
Three-dimensional response surface plot presenting the interaction effect between concentrations of carbopol 940 (X_1_) and percentage of KCME (X_2_) on hardness (Y_1_).

According to the model of Y_1_, the *p*-value is 0.0003, and *R*^2^ is 0.9464, implying that this model could accurately interpret approximately 94.64% of the hardness values. Quadratic terms of both concentrations of carbopol 940 (X_1_) and the percentage of KCME (X_2_) did not exhibit a significant impact on hardness, thereby choosing the two-factor interaction (2FI) model. In the case of the nonsignificant main X_1_ effect (*p*-value 0.9072), this term was also included in the model owing to the significant interaction effect of X_1_X_2_ (*p*-value 0.0144). From the Y_1_ model, the percentage of KCME (X_2_) showed the strongest negative influence on hardness (*p* < 0.0001), implying a decrease in hardness value with respect to an increase in KCME. In addition, the interaction effect of X_1_ and X_2_ is illustrated in Figure 5. The highest hardness value could be achieved at the highest concentration of carbopol (X_1_) and the lowest percentage of KCME (X_2_). Similarly, no significant difference in hardness value was indicated by the variation of X_1_ in the experimental range.

Moreover, the adhesiveness values (Y_2_) could be interpreted using the following linear model below:

Y_2_ = 9.49 + 5.65X_1_ − 18.82X_2_

According to the model *R*^2^ of 0.9010, in which the significant main effects of X_1_ and X_2_ were included, 90.10% of adhesiveness can be accurately predicted. As shown in Table 3, the *p*-value of the model is 0.0003, implying a significant model describing the correlation between independent variables and adhesiveness. Two significant main effects, including the concentration of carbopol 940 (X1) (*p*-value 0.0161) and the percentage of KCME (*p*-value 0.0002), are included in the final model. In the final model, the concentration of carbopol 940 (X_1_) was positively proportional to the variation of adhesiveness, whereas the percentage of KCME (X_2_) was negatively proportional. Moreover, the influence of X_2_ was superior to that of X_1_, which corresponded to that of the hardness model.

The desirable attributes of mucoadhesive formulation gels include ease of removal from the primary package, ease of application, and retention of the product at the application site without disintegration [60]. The hardness is considered a necessary force to provide the deformation of gels [60]. This parameter implies the applicability of the gel to the desired site. In addition, high adhesiveness enhances the stickiness of the formulation and prolongs the attachment even in the absence of mucoadhesive bonds. Our results revealed that both the hardness and adhesiveness of the formulation containing carbopol 940 were decreased owing to the increase in KCME content, which corresponded to that of the study of Špaglová et al. [61]. Carbopol is one of the polyacrylic acid gelling agents that forms a network of cross-linked polymer chains that stabilize the gel. By increasing the concentration of carbopol, a more tightly cross-linked network of polymer chains is generated, conferring higher hardness to the formulation. This also contributes to the adhesiveness of the mucoadhesive gel by providing a better bind force for the gel to the applied surface [62]. Owing to the increase in KCME content, the final concentration of carbopol in the formulation was proportionally reduced, thereby decreasing both hardness and adhesiveness. 

**Figure 6:**
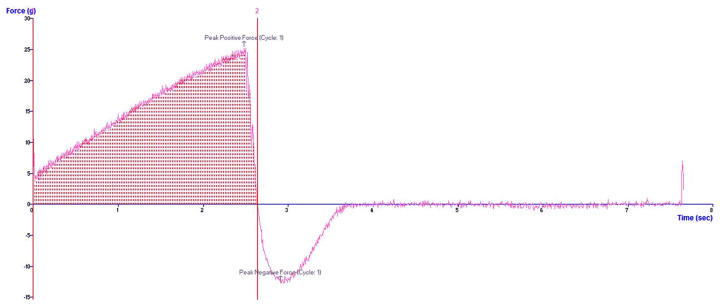
Texture profile analysis of optimized KCMEG.

According to the study of Basu et al., hardness less than 28 g and adhesiveness greater than 30 g.s were considered the optimum conditions for the transnasal formulation [63]. Therefore, these targets were numerically optimized to generate the optimized formulation, which could be fabricated using a concentration of carbopol 940 of 2% and a percentage of KCME of 25%, which was observed in hardness and adhesiveness as shown in Figure 6. The experimental and predicted values generated by the models are shown in Table 4. In the case of the model of hardness, the prediction error calculated by the difference between experimental and predicted values of less than 5% implied the good accuracy of the verified model. However, in the model of adhesiveness, the difference between experimental and predicted values was higher than 5%, indicating the unacceptable accuracy of this model.

### 2.6. In Vitro Mucoadhesive Study

The mucoadhesive potential of optimized KCMEG was evaluated using an in vitro method, and the result is tabulated in Table 2. The retention times shown by KCME and KCMEG were 2.32 ± 0.08 and 9.67 ± 0.13 min, respectively. The retention time on the agar plate shown by KCMEG was significantly higher than that shown by KCME. Thus, the developed mucoadhesive preparation, KCMEG, was hypothesized to be able to increase the contact time between the dosage form and mucosal layers of nasal cavities, which could be attributed to the presence of carbopol 940, which agreed with another report [58]. Moreover, related literature reported that the retention time of formulation on the mucosal membrane affects drug diffusion across the nasal mucosa and reduces nasal mucociliary clearance, thereby affecting brain delivery [59]. Nasal mucociliary clearance is a crucial mechanism responsible for eliminating foreign particles adhered to the mucus surface. Consequently, drugs administered via the transnasal route may be cleared by this mechanism, leading to low drug absorption into the brain [6,64]. To overcome mucociliary clearance, drugs need to be designed to strongly interact with the nasal mucosa, thereby increasing the contact time and enhancing drug absorption. Therefore, an effective transnasal formulation should extend the residence time on the nasal mucosa. Furthermore, the study conducted by Khan et al. confirmed a significant increase in drug accessibility to the blood and brain when administered intranasally as a mucoadhesive formulation compared to a nasal solution [46]. It is worth noting that conventional liquid formulations are typically eliminated from the nasal cavity within approximately 15 min of being deposited on the mucus surface. Therefore, the mucoadhesive properties of formulations should be investigated concurrently with drug diffusion to the brain [65].

### 2.7. Nasal Cilio-Toxicity

**Figure 7:**
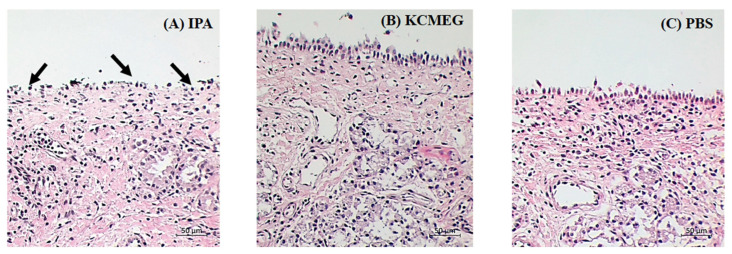
Optical microscopic images (20×) of nasal mucosa treated with: (**A**) positive control—isopropyl alcohol; (**B**) KCMEG and (**C**) negative control—PBS pH 6.4 where the arrows indicate damage of nasal tissue.

Nasal ciliotoxicity was studied to evaluate the toxic effect of the sample used for nasal mucosa formulations. The porcine nasal mucosa was treated with isopropyl alcohol, a mucociliary toxic agent, optimized KCMEG, and a negative control (PBS pH 6.4). The porcine nasal tissue treated with isopropyl alcohol destroyed the epithelium layer with damage to internal nasal tissues (arrows), as shown in Figure 7A. In contrast, those treated with KCMEG and PBS showed an intact epithelium layer without tissue damage (Figure 7B,C), indicating the safety of excipients used in the formulation of KCMEG in this study. Our results agreed with other reports on the toxicity of oleic acid, tween 80, and carbopol, which are the major components of KCMEG [42,66], demonstrating the safety profile of KCMEG for transnasal administration. In vitro techniques are frequently a useful screening method for identifying substances that may have undesirable impacts on the nasal mucosal structure [67]. To demonstrate a safety profile for human use, it is essential to investigate the effect of a nasal drug formulation at biopharmaceutically and therapeutically related concentrations Further, the effects of long-term use of nasal formulations in animals and humans should be determined for accuracy and reliability in evaluations of the potential side effects [67].

However, the safety profile is insufficient to explain the efficacy, biodistribution, and pharmacokinetics of the formulation when delivered into the brain. Thus, it is necessary to perform in vivo studies to verify the efficacy of the formulation. Interestingly, the literature has reported that nanoformulation can enhance the stability, bioavailability, bioaccumulation, and pharmacokinetic parameters of curcumin compared to free curcumin [68]. In addition, Zang et al. formulated a curcumin/hydroxypropyl-β-cyclodextrin inclusion complex and reported that the plasma and brain concentrations of the formulation were higher than those in the control group after intranasal administration [69]. Moreover, Li et al. reported that curcumin-lactoferrin nanoparticles demonstrated excellent brain accumulation, prolonged elimination half-life, and improved bioavailability of curcumin after intranasal administration [70]. Based on this information, it indicates the strong performance of nanoformulations to improve the pharmacokinetics and efficacy of curcumin. Hence, it can be hypothesized that KCME and KCMEG could improve the pharmacokinetics and efficacy of curcumin. However, in vivo tests are essential to verifying this hypothesis.

## 3. Conclusions

In this present investigation, KLVFF conjugated curcumin microemulsion (KCME) was prepared using water titration. In vitro anticholinesterase activity and anti-amyloid beta aggregation demonstrated the opportunity for KCME to serve as a candidate to treat AD through the transnasal route. Based on hardness and adhesiveness tests conducted with CCD, the formulation contains 2% carbopol 940 and 25% KCME, which was considered an optimized microemulsion-based hydrogel in this study. KCMEG demonstrated suitable mucoadhesive properties with a safety profile for the transnasal delivery of curcumin. However, in vivo studies and biodistribution studies are required to display and confirm the potential of KCMEG for the nasal transport pathway to the brain directly. In addition, a detailed animal study followed by clinical trials is required to establish the clinical safety and efficacy of this formulation.

## 4. Materials and Methods

### 4.1. Materials

Pluronic F-127^®^ was a gift from BASF Chemical Company (St. Louis, MO, USA), and 1-Ethyl-3-[3-dimethylaminopropyl] carbodiimide hydrochloride (EDC), N-hydroxysulfosuccinimide (sulfo-NHS), tetrahydrofuran (THF), dimethylaminopyridine (DMAP), acetylthiocholine iodide (ATCI), acetylcholinesterase (AChE), and 5,5′-dithio-bis-2-nitrobenzoic acid (DTNB) were purchased from Sigma Aldrich (St. Louis, MO, USA). Ethanol, methanol, and carbon tetrachloride were obtained from Sigma-Aldrich (Steinheim, Germany). Curcumin, dimethylsulfoxide (DMSO), tris-HCl, glycine, and thioflavin T were obtained from Merck (Darmstadt, Germany). Aβ_1–42_ and KLVFF peptides were synthesized and purified by Pepmic (Suzhou, China). Monobasic potassium phosphate was purchased from RCI Labscan (Bangkok, Thailand). Sodium hydroxide was obtained from Kemaus (Cherrybrook, New South Wales, Australia). Carbopol 940 was purchased from Chemipan (Bangkok, Thailand). Galantamine was purchased from Calbiochem (San Diego, CA, USA). Polysorbate 80 (Tween 80) and triethanolamine were purchased from UnionSci (Chiang Mai, Thailand).

### 4.2. Preparation of Curcumin-Loaded KLVFF-Pluronic F127 ME (KCME)

In our previous study, we successfully developed curcumin-loaded KLVFF-Pluronic F127 ME (KCME). Briefly, a pseudo-ternary phase diagram was constructed to determine the suitable component of the microemulsion. Surfactants (a mixture of Tween 80 and KLVFF-Pluronic F127 in the ratio 1:1) and several co-surfactants (ethanol, PEG 400, and PG), namely, S_mix_, were mixed in different ratios (1:1, 2:1, 3:1, 4:1) to prepare ME according to the area existing in the phase diagram. For the construction of the phase diagram, the mixtures of oil, S_mix_, and water at different ratios (9:1, 8:2, 7:3, 6:4, 5:5, 4:6, 3:7, 2:8, and 1:9) were formulated using a titration method under continuous stirring until a transparent ME was formed. The determination of the ME region was performed by visual observation of the turbidity. The samples were classified as ME when they appeared visually as clear liquids. Pseudo-ternary phase diagrams were drawn using SigmaPlot software version 11.0 (Systat Software, Inc., Chicago, IL, USA), and the areas of the ME regions were measured by ImageJ 1.47v software (National Institutes of Health, Bethesda, MD, USA). According to our previous result, the suitable formulation consisted of oleic acid as the oil phase, a mixture of Tween 80 and KLVFF-Pluronic F127 in the ratio 1:1 (surfactant), ethanol (co-surfactant) as a mixture of surfactant and co-surfactant (S_mix_), and water as the aqueous phase. We reported that the most suitable ME component was 15:80:5 for the oil:S_mix_:water ratio, respectively [71] This microemulsion component exhibited optimal results and was chosen to create KCME in this study. Curcumin (0.9 mg/mL) was dissolved in the oil phase by adding the required amount of S_mix_ and water and stirring to create a clear, yellow, and transparent dispersion. The percentage of transmittance, globule size, zeta potential, and pH of KCME were all examined.

### 4.3. Physicochemical Characterization of ME and Microemulsion-Based Hydrogel

Dynamic light scattering (SZ-100, Horiba, Japan) was used to determine the average droplet size and polydispersity index (PDI) of KCME. All determinations were made in triplicate. The transmittance of KCME was measured using a UV spectrophotometer (UV2600i, Shimadzu, Japan) at 650 nm using pure water as a reference [72]. The pH value of KCME was assessed using a digital pH meter (pH meter, Metrohm, Herisau, Switzerland). For the pH of the KCMEG, 1.0 g of KCMEG was dispersed in 100 mL of distilled water, and then the pH was measured after storing it for 2 h [73]. The device was first standardized using pH 4 and 7 buffers. A spreadability test involved pushing 0.5 g of each prepared formula between two glass slides and waiting for about 5 min until no more spreading was anticipated. The diameter of the circle was measured and used as a result for spreadability [74].

### 4.4. Fourier Transform Infrared Spectroscopy (FTIR)

To identify the distinctive peaks of materials that would emerge based on their chemical structure, curcumin, KMEG, and KCMEG were measured using FTIR (470FT-IR Nicolet Nexus FTIR spectrometer, Thermo Electron Corporation, Madison, WI, USA) [75]. The FTIR spectra were operated in the range of 4000 to 400 cm^−1^. The FTIR spectra of the sample were determined using Multi-Bounce HATR Kits (SMART, Thermo Electron Corporation, Madison, WI, USA).

### 4.5. Anticholinesterase Activity

Ellman’s method was used to perform the enzyme inhibitory action [76]. Acetylcholinesterase (AChE) was employed as an enzyme and ATCI as the substrate, respectively. Specifically, 50 µL of 50 mM Tris-HCl buffer pH 8.0, 25 µL of 1.5 mM ATCI, 125 µL of 3 mM DTNB, and 25 µL of KCME in Tris-HCl buffer containing 10% methanol were mixed in the appropriate amounts. After that, 25 µL of 0.25 U/mL AChE was added, and a microplate reader was used to measure the kinetic reaction spectrophotometrically every 2 min at 415 nm (SPECTROstar^®^ Nano, BMG LABTECH, Ortenberg, Germany). The cholinesterase activity of KCME (0.00015–0.005 mg/mL) was assessed using a Tris-HCl solution containing 10% methanol as a negative control. Galantamine hydrobromide (0.000006–0.6 mg/mL) was used as a positive control. The tests were carried out in triplicate. The enzymatic reaction rate was calculated from the slope of the absorbance versus time plot. The equation below was used to determine the enzyme inhibitory activity.

$$\%\;\mathrm{Inhibition}\;=\;\left(\frac{V_{s}-V_{b}}{V_{b}}\right)\;\times \;100$$

where V_s_ is the reaction rate of the sample and V_b_ is the reaction rate of the blank. Data were fitted with nonlinear regression to determine IC_50_ using GraphPad Prism, Version 8.0 software.

### 4.6. Anti-Aggregation of Beta Amyloid

The thioflavin T assay was conducted to investigate the inhibition of Aβ aggregation [77,78]. Nine microliters of 25 µM Aβ solution were mixed with the samples and incubated for 48 h with no agitation. DMSO, which does not affect the assay, was used as the solvent. After an incubation period, 200 µL of a 5 µM thioflavin-T solution in glycine was added. Fluorescence intensity was measured using SpectraMax i3 (Molecular Device, San Jose, CA, USA) with excitation and emission wavelengths at 446 and 500 nm, respectively. The percentage of inhibition was calculated using the formula below.

$$\%\;\mathrm{Inhibition}\;=\left[1-\frac{\left(F_{S}-F_{\mathrm{SB}}\right)}{\left(F_{\mathrm{AB}}-F_{\mathrm{RB}}\right)}\right]\times 100$$

where F_S_ is the fluorescent intensity of the sample, F_SB_ is the fluorescent intensity of the sample blank, F_AB_ is the fluorescent intensity of the free aggregation control, and F_RB_ is the fluorescent intensity of the reagent blank. IC_50_ was determined by nonlinear regression analysis using GraphPad Prism Software, Version 8.0.

### 4.7. Optimization of Curcumin-Loaded KLVFF-Pluronic F127 ME-Based Hydrogel (KCMEG) by CCD

Response surface methods have been ubiquitously employed to create the most desirable formulations based on the statistical approach [47]. Based on influencing factors, the effects of concentrations of carbopol 940 (X_1_) and percentage of KCME (X_2_) on response variables including hardness (Y_1_) and adhesiveness (Y_2_) were optimized using CCD, at which five levels of X_1_ and X_2_ were varied as shown in Table 5. Subsequently, 13 experimental runs were created and conducted, as shown in Table 6. The studied responses were statistically analyzed using analysis of variance (ANOVA) through Design-Expert^®^ Software (Version 10.0; Stat-Ease Inc., Minneapolis, MN, USA) to evaluate the significance of main and interaction effects together to generate the mathematical equation explaining the correlation between factors and responses in terms of a second-order polynomial equation, as shown below. The terms that were included in the final model were selected based on their level of significance, where a *p*-value less than 0.05 indicates statistical significance.

$$Y_{i}=\beta_{0}+\sum \beta_{i}X_{i}+\sum \beta_{ii}X_{ii}^{2}+\sum \beta_{ij}X_{i}X_{j}$$

where Y_i_ is a response variable; β_0_ is a constant; β_i_, ii, and β_ij_ are regression coefficients of the main quadratic equation and interaction terms, respectively. The desirable values of responses were then predicted according to the significant mathematical model (*p* < 0.05) with a non-significant lack of fit and an acceptable coefficient of determination (*R*^2^), which was at least 0.80. A non-significant term (*p* > 0.05) was excluded from the final model. Unless a quadratic or interaction term of the variable was significant (*p* < 0.05), the linear term was then kept in the final reduced model.

### 4.8. Optimization and Verification

To determine the interaction effect of the independent variables affecting the response variables, the response surface in terms of the 3D graph was generated according to the final reduced model of each response. While the interaction effect of two variables was shown as a variation of response values within the experimental range, another independent variable was the constant point. Additionally, numerical optimization was employed to enhance the target values of the independent variables with the highest desirability. Verification of the final reduced models was then conducted by comparing the actual experimental and theoretical predicted values in terms of percentage prediction error, which should be no more than a 5% difference to accept the verified statistical models.

### 4.9. In Vitro Mucoadhesion Study

The mucoadhesive potential of KCME and KCMEG was evaluated using an in vitro method reported by Nakamura et al. and Bachhav et al. [74,79]. Each 50 mg of KCME and KCMEG was placed at the center of an agar plate (1%, *w*/*w* in pH 6.4 phosphate buffer). After 5 min, the agar plate was attached to a USP disintegration test apparatus and moved up and down in pH 6.4 phosphate buffer at 37 ± 1 °C. The formulation on the plate was immersed in the solution at the lowest point and out of the solution at the highest. The residence time of the KCME and KCMEG on the plate was investigated visually. The residence time was noted and recorded at the time point at which the formulation unstuck on agar.

### 4.10. Morphological Analysis (SEM)

The morphology of the optimized KCMEG was observed by SEM (Tabletop TM4000Plus, HITACHI, Japan). KCMEG was deposited onto a carbon-conductive adhesive carbon tape (Nisshin EM, Tokyo, Japan), taped to an aluminum grid, and dried in air. The examinations were performed without any conductive coating [80]. Then KCMEG was conducted in a high vacuum using a secondary electron detector at an accelerating voltage of 5 kV.

### 4.11. Nasal Cilio-Toxicity Study

Damage to the cilia may contribute to the problem of formulation for use through the intranasal route. Therefore, the toxicity of KCMEG on nasal cilia was investigated in this study. The protocol for the nasal ciliotoxicity test followed the method described by Pailla et al. [81]. Freshly excised porcine nasal mucosa was obtained from the slaughterhouse and immediately soaked in phosphate buffer (pH 6.4). The cartridge was gently taken out to isolate the nasal mucosa. Each piece of the porcine nasal mucosa was mounted on a Franz diffusion cell for 2 h with isopropyl alcohol as the positive control, PBS pH 6.4 as the negative control, and KCMEG. After that, each piece of mucosa was thoroughly cleaned with PBS (pH 6.4) and allowed to soak overnight in a 10% *v*/*v* formalin solution. Each mucosa was preserved in paraffin blocks, and fine sections were taken (7 mm thick) and stained with eosin and hematoxylin. The produced slides were examined using an inverted microscope (Motic, AE2000, Quebec, Canada) at a 20× magnification to assess any nasal mucosa damage; intact nasal mucosa is the criterion for evaluation of the safety profile of the formulation [21]. The protocol for the use of cadavers was approved by the Animal Care and Use Committee, Faculty of Veterinary Medicine, Chiang Mai University, Thailand (FVM-CMU-ICUC Ref. No. R5/2563).

### 4.12. Statistical Analysis

All data are presented as mean ± SEM, n = 3 experiments. The significance of the difference between the two groups’ means was assessed using the T-test. A one-way analysis of variance (ANOVA) followed by the Newman-Keuls post hoc test was used to conduct statistical analysis to determine the significance of any differences. A value of *p* < 0.05 was regarded as statistically significant in each case.

## Figures and Tables

**Table 1:** Physicochemical parameters of KCME.

Formulation	Globule Size (nm)	PDI	Zeta Potential (mV)	%T	pH
KCME	70.8 ± 3.4	0.409 ± 0.048	−0.07 ± 0.74	97.03 ± 0.01	5.80 ± 0.02

**Table 2:** Spreadability and mucoadhesion properties of KCME and KCMEG.

Formulation	Diameter of Spread Area (cm)	Retention Time (Min)
KCME	3.22 ± 0.17	2.32 ± 0.08
KCMEG	0.90 ± 0.15	9.67 ± 0.13

**Table 3:** *R*-squared (*R*^2^), adjusted *R*^2^, probability values, and the significance probability (*p*-value, *F*-value) of regression coefficients in the final reduced models.

	Variable	Hardness (Y_1_)		Adhesiveness (Y_2_)	
		*F*-Value	*p*-Value	*F*-Value	*p*-Value
Main effects	X_1_	0.0148	0.9072	9.93	0.0161 ^a^
	X_2_	87.91	<0.0001 ^a^	53.78	0.0002 ^a^
Quadratic effects	X_1_^2^	-	-	-	-
	X_2_^2^	-	-	-	-
Interaction effects	X_12_	11.60	0.0144 ^a^	-	-
*R* ^2^		0.9464		0.9010	
Adjusted *R*^2^		0.9197		0.8727	
*p*-value of model		0.0003 ^a^		0.0003 ^a^	

^a^ indicated not significant at *p* > 0.05.

**Table 4:** Experimental and predicted values in terms of hardness and adhesiveness of the optimized formulation.

Response	Experimental Value	Predicted Value	Prediction Error (%)
Hardness (g)	25.360	25.277	0.33%
Adhesiveness (g.s)	38.98	33.874	15.07%

**Table 5:** Code levels and actual values of influencing factors.

Factors	Code Levels and Actual Values				
	Axial (−α)	Low (−1)	Center (0)	High (+1)	Axial (+α)
X_1_	0.19	0.50	1.25	2.00	2.31
X_2_	14.64	25	50	75	85.36

X_1_: Concentrations of carbopol 940; X_2_: percentage of KCME.

**Table 6:** Experimental matrix of the central composite design and the experimental data obtained for the response variables studied; hardness (Y_1_) and adhesiveness (Y_2_).

Run	Independent Variable		Response Variable	
	X_1_	X_2_	Hardness (Y_1_: g)	Adhesiveness (Y_2_: g.s)
1	1.25	50	9.981	16.282
2	1.25	50	7.059	10.479
3	0.50	25	10.467	17.208
4	2.31	50	7.546	11.439
5	1.25	50	6.329	8.982
6	1.25	50	6.086	9.409
7	1.25	14.64	26.655	39.575
8	0.19	50	7.181	0.097
9	2.00	75	N/A	N/A
10	1.25	50	6.451	11.179
11	2.00	25	23.247	34.523
12	1.25	85.36	N/A	N/A
13	0.50	75	N/A	N/A

N/A = not analyzed.

## Data Availability

Not applicable.
